# Prevalence and risk factors for acute kidney injury among trauma patients: a multicenter cohort study

**DOI:** 10.1186/s13054-018-2265-9

**Published:** 2018-12-18

**Authors:** Anatole Harrois, Benjamin Soyer, Tobias Gauss, Sophie Hamada, Mathieu Raux, Jacques Duranteau, for the Traumabase® Group, Olivier Langeron, Catherine Paugam-Burtz, Romain Pirracchio, Bruno Riou, Guillaume de Saint Maurice, Xavier Mazoit

**Affiliations:** 1 Université paris Sud, Université Paris Saclay, Department of Anesthesiology and Critical Care, Assistance Publique-Hopitaux de Paris (AP-HP), Bicêtre Hopitaux Universitaires Paris Sud, 78 rue du Général Leclerc, 94275 Le Kremlin Bicêtre, F-94275 Le Kremlin Bicêtre, France; 2 Hôpitaux Universitaires Paris Nord Val de Seine, Department of Anesthesiology and Critical Care, AP-HP, Beaujon, 100 avenue du Général Leclerc, 92110 Clichy, France; 3 Sorbonne Université, INSERM, UMRS1158 Neurophysiologie Respiratoire Expérimentale et Clinique; AP-HP, Groupe Hospitalier Pitié-Salpêtrière Charles Foix, Département d’Anesthésie Réanimation, Paris, France; 4 Hôpital de Beaujon, Anesthésie-Réanimation, 100, boulevard du Général Leclerc, 92110 Clichy, France; 5 Hôpital Pitié-Salpétrière, Anesthésie-Réanimation, 47-83 Boulevard de l’Hopital, 75013 Paris, France

**Keywords:** Acute kidney injury, Trauma, Rhabdomyolysis, Hemorrhagic shock, Renal failure, Organ failure

## Abstract

**Background:**

Organ failure, including acute kidney injury (AKI), is the third leading cause of death after bleeding and brain injury in trauma patients. We sought to assess the prevalence, the risk factors and the impact of AKI on outcome after trauma.

**Methods:**

We performed a retrospective analysis of prospectively collected data from a multicenter trauma registry. AKI was defined according to the risk, injury, failure, loss of kidney function and end-stage kidney disease (RIFLE) classification from serum creatinine only. Prehospital and early hospital risk factors for AKI were identified using logistic regression analysis. The predictive models were internally validated using bootstrapping resampling technique.

**Results:**

We included 3111 patients in the analysis. The incidence of AKI was 13% including 7% stage R, 3.7% stage I and 2.3% stage F. AKI incidence rose to 42.5% in patients presenting with hemorrhagic shock; 96% of AKI occurred within the 5 first days after trauma. In multivariate analysis, prehospital variables including minimum prehospital mean arterial pressure, maximum prehospital heart rate, secondary transfer to the trauma center and data early collected after hospital admission including injury severity score, renal trauma, blood lactate and hemorrhagic shock were independent risk factors in the models predicting AKI. The model had good discrimination with area under the receiver operating characteristic curve of 0.85 (0.82–0.88) to predict AKI stage I or F and 0.80 (0.77–0.83) to predict AKI of all stages. Rhabdomyolysis severity, assessed by the creatine kinase peak, was an additional independent risk factor for AKI when it was forced into the model (OR 1.041 (1.015–1.069) per step of 1000 U/mL, *p* < 0.001). AKI was independently associated with a twofold increase in ICU mortality.

**Conclusions:**

AKI has an early onset and is independently associated with mortality in trauma patients. Its prevalence varies by a factor 3 according to the severity of injuries and hemorrhage. Prehospital and early hospital risk factors can provide good performance for early prediction of AKI after trauma. Hence, studies aiming to prevent AKI should target patients at high risk of AKI and investigate therapies early in the course of trauma care.

**Electronic supplementary material:**

The online version of this article (10.1186/s13054-018-2265-9) contains supplementary material, which is available to authorized users.

## Background

Organ dysfunction remains the third leading cause of death in trauma patients, after hemorrhage and traumatic brain injury [1]. Among organ failure after trauma, AKI is common, with reported incidence up to 50% and has been independently associated with prolonged hospital length of stay and mortality [2, 3].

Severe trauma triggers initial AKI risk factors including hemorrhage, rhabdomyolysis, traumatic inflammation and leads to second hits due to emergency surgery or infections that may cause additional renal disorders resulting in renal function impairment. Identifying AKI risk factors after trauma is essential to establish a strategy aiming to prevent AKI and its related complications. Previous studies reporting AKI incidence after trauma focused either on medical history [4, 5], hemodynamic variables [6, 7], type of trauma [8] or rhabdomyolysis [9] as potential risk factors for AKI, but including all of them would provide a more complete overview of the renal aggression associated with renal dysfunction. Moreover, the prehospital period is hardly taken into account, yet it is a time during which renal aggression is likely to occur (i.e. hypotension, hypoxemia). In addition, most studies included patients who were admitted to the intensive care unit (ICU) after trauma, subsequently selecting those who are the most severely injured, even though attention should also be paid to patients with moderate injuries.

The objectives of this study were (1) to report the prevalence of AKI, (2) to describe risk factors associated with AKI and (3) to explore whether AKI is independently associated with mortality in a multicenter cohort of trauma patients whose characteristics and physiological variables are prospectively collected in a research registry.

## Methods

We conducted a retrospective multicenter observational study in three French regional level-1-designated trauma centers between May 2011 and July 2014. The three trauma centers progressively joined the TraumaBase registry between 2011 and 2012 (http://www.traumabase.eu/fr_FR). Clinical, biological and anamnestic data on every patient admitted to each of these three centers were prospectively collected in the database. The TraumaBase® group obtained approval for this study, including waived informed consent from the Institutional Review Board (Comité pour la Protection des Personnes, Paris VI-Pitié-Salpêtrière, France). The database was approved by the Advisory Committee for Information Processing in Health Research (Comité Consultatif sur le Traitement de l’Information en matière de Recherche dans le Domaine de la Santé, CCTIRS 11.305 bis), and the French National Commission on Computing and Liberty (Commission Nationale Informatique et Liberté, CNIL 911461).

### Study population

All trauma patients older than 16 years admitted to the three participating centers, as primary or secondary (from another facility within 48 h after trauma) transport were included in the study. Beaujon, Pitié-Salpêtrière and Bicêtre University Hospitals are three trauma centers in the Paris region. They manage the care of approximately 60% of patients with severe trauma in the Paris region (12 million inhabitants). The Paris emergency medical system and trauma management have been described elsewhere [10]. Patients suspected of severe trauma (according to the presence of at least one Vittel triage criterion as assessed by the physician on scene, see Additional file 1) are directly admitted into ICU in any of the the participating trauma centers. Our records show that the systematic admission of patients suspected of severe trauma into ICU involves a subgroup of patients with mild to moderate injuries who are not usually included in studies on trauma AKI [11].

Data collected in the prehospital setting included blood pressure, heart rate, peripheral oxygen saturation, trauma characteristics, Glasgow Coma Scale (GCS) score and vasopressor use. On arrival at the hospital, hemodynamic, respiratory and neurologic variables were recorded. Blood was immediately sampled for blood typing and analysis that usually (though not always) included blood gas, lactate, creatine kinase (CK) and coagulation tests. Maximum CK was the highest level of CK recorded during the ICU stay. The clinical severity was assessed using the following severity scores: Simplified Acute Physiology Score II (SAPS II) and Sequential Organ Failure Assessment (SOFA). The severity of traumatic injuries was assessed using the Abbreviated Injury Scale (AIS) 1998 version and the Injury Severity Score (ISS). The trauma-related injury severity score (TRISS) was also determined [12, 13]. Considerable renal trauma was defined by renal AIS ≥ 3, which includes (1)renal parenchyma injuries ranging from a laceration of at least 1 cm depth to a complete renal fracture, and/or (2) renal vessel injury and/or (3) collecting-duct injuries. ICU and hospital length of stay and ICU mortality were reported. Of note, every patient underwent contrast-enhanced whole-body computed tomography (CT) in the initial phase of care.

The TraumaBase registry included all the patients admitted to the study centers. Data are entered manually by dedicated research technicians. Algorithms for consistency and coherence are integrated into the database structure. A core dataset of 35 variables for which the collection of data is considered mandatory was established prior to data collection in the registry. Completeness of data was monitored and regularly checked in each center. Data monitoring is performed by a central administrator.

In the database, hemorrhagic shock is defined by the transfusion of at least four units of packed red blood cells (RBC) within the first 6 h [14, 15]. Traumatic brain injury (TBI) is defined by at least one traumatic lesion on the initial cerebral CT scan.

### Renal function assessment

Renal function was assessed with serum creatinine variations according to the classification of risk, injury, failure, loss, end-stage of kidney disease (RIFLE) [16] and the worst RIFLE stage over ICU stay was recorded in the registry. No data on renal function were collected after discharge from ICU. Trauma patients’ baseline creatinine measurement is rarely available so we chose the reference as the lowest value of plasma creatinine during the first 5 days of hospitalization [17–20]. This methodology was recently reported to be more accurate than the creatinine value back-calculated using the Modified Diet in Renal Disease formula (MDRD) formula for a glomerular filtration rate of 75 mL∙min^− 1^ per 1.73 m^2^ in a population of young trauma patients [20]. We defined early AKI as AKI occurring during the first 5 days after admission and late AKI as AKI occurring after 5 days [4].

### Statistical analysis

Quantitative variables were expressed as mean (SD) or median (25th–75th interquartiles) according to their distribution and categorical variables were expressed as counts (proportions). Prevalence of each AKI severity stage was reported in the whole cohort and in three subpopulations commonly reported in the literature: (1) severely injured patients presenting with an ISS ≥ 16 [21], (2) trauma patients who required more than one packed RBC concentrate during their ICU stay [22, 23] and (3) trauma patients with hemorrhagic shock [24]. In the next step, we created a binary outcome variable that had either a value of 0 when there was no AKI or a value of 1 when there was AKI stage R, I or F. We also created a binary outcome variable that had either a value of 0 when there was no AKI or AKI stage R or a value of 1 when there was AKI stage I or F. This relies on the fact that AKI stage R mostly occurs within the first 24 h after trauma, which renders the predictive model for AKI stage R less relevant from a chronological perspective when including risk factors that require 6-h transfusions (hemorrhagic shock).

Risk factors for stage I or F AKI (or for AKI all stages) were evaluated in univariate analysis (*t* test for Gaussian variables, chi-square test for proportions and Mann Whitney test for non-normally distributed variables). We selected those factors significantly associated with AKI in the univariate analysis (*p* < 0.20) with two other clinical relevant factors: age, which is a well-known risk factor for AKI in ICU and direct/secondary transfer to a trauma center as a surrogate of time to specialized care, which is elsewhere reported to be linked to outcome [25]. Spearman correlation coefficients were computed to evaluate collinearity (*r* > 0.8). If variables were correlated, we chose the most relevant one from a clinical/physiological perspective. Candidate variables were entered into stepwise multivariate logistic regression using a backward selection model. To better assess the performance of the predictive model, bootstrapping (10,000 resampling) analysis was used to estimate the odd ratios (OR) and 95% confidence intervals (CI). As an internal validation, bootstrapping provides robust estimates with low bias by addressing optimism and overfitting [26, 27]. The model calibration was assessed using the Hosmer-Lemeshow statistic and the discrimination by reporting the area under the receiver operating characteristic curve (AUC-ROC). First-order interactions were investigated.

CK peak was not included in the predictive multivariate models because it is a late variable that is not relevant to predict AKI. However, as a marker of rhabdomyolysis severity, it was included in a second multivariate analysis to explore whether rhabdomyolysis was independently associated with AKI or not.

We built ROC curves for various thresholds of variables associated with AKI. Sensitivity, specificity, positive and negative predictive values (PPV and NPV), positive and negative likelihood ratios (PLR and NLR) and calculation of the AUC-ROC were reported for each variable. The best threshold was defined as the value maximizing the Youden index (sensitivity + specificity – 1). To investigate the association between AKI and ICU mortality, the factor AKI was entered into a stepwise logistic regression model with other factors associated with the outcome (death) in the univariate analysis. Missing data were not replaced and only complete cases were included in the multivariate models. Proportions of missing data and characteristics of patients with missing data were reported for each model. The two-sided level of significance was fixed at 5%. Results were analyzed using R open source software 3.1.1 (https://www.r-project.org/) (The R Foundation for Statistical Computing, Vienna, Austria).

### Sample size calculation

The prevalence of severe AKI (stage I or F) is variable in cohort of trauma patients, with reported percentages ranging from 1 to 26% [2–6, 9, 17, 18]. Moreover, we have no estimate of AKI prevalence in our research database. This precludes calculation of a sample size that would accurately provide the required number of events established using previously published rules (at least 100 events) to build risk prediction models [27]. For this reason, we included all patients available in the database over the study period (*n* = 3111) thereby expecting to provide robust estimates even in the case of severe AKI prevalence as low as 3%.

## Results

There were 3488 trauma patients recorded in our database from June 2011 to July 2014. Data on renal function were available for 3111 patients. Characteristics of the 377 excluded patients are reported in Additional file 2. The characteristics of the overall cohort are presented in Table 1. Trauma patients were young, 38 (18) years, with a median ISS of 14 (9–25) and had predominantly blunt trauma (91%): 31% of patients presented with TBI. Hemorrhagic shock was reported in 11.4% of patients. The overall mortality was 10.8%.

**Table 1:** General, physiologic and injury severity characteristics of the patients in the overall population and in both subgroups of acute kidney injury (AKI) severity

Characteristics	Whole cohort	No early AKI or AKI stage R	Early AKI stage I or F	*p* value
	*n* = 3111	*n* = 2934	*n* = 177	
General characteristics				
Age, years	38 ± 18	38 ± 17	39 ± 17	0.58
Male sex, *n* (%)	2428 (78.0)	2284 (77.8)	144 (81.3)	0.27
Direct transfer to trauma center, *n* (%)	2589 (83.2)	2441 (83.2)	148 (83.6)	0.70
SAPS II	21 [11–38]	20 [11–36]	47 [31–65]	< 0.001
SOFA 24 h	2 [0–6]	1 [0–6]	9 [5–13]	< 0.001
ISS	14 (9–25)	13 [8–24]	33 [21–41]	< 0.001
Head and neck AIS	1 [0–3]	0 [0–3]	2 [0–3]	0.02
Abdomen AIS	0 [0–2]	0 [0–2]	2 [0–3]	< 0.001
Thorax AIS	0 [0–3]	0 [0–3]	3 [2–4]	< 0.001
Extremities and pelvis AIS	1 [0–3]	1 [0–2]	2 [0–3]	< 0.001
Blunt trauma, *n* (%)	2835 (91.1)	2668 (90.9)	167 (94.3)	0.11
MVA, *n* (%)	652 (21)	610 (20.8)	42 (23.7)	–
Motorbike, *n* (%)	741 (23.8)	705 (24)	36 (20.3)	–
Fall, *n* (%)	890 (28.6)	831 (28.3)	59 (33.3)	–
Pedestrian, bicycle, *n* (%)	362 (11.6)	338 (11.5)	24 (13.6)	–
Other, *n* (%)	190 (6.1)	184 (6.3)	6 (3.4)	–
Penetrating trauma, *n* (%)	276 (9)	266 (9)	10 (6)	0.11
Gunshot wound, *n* (%)	87	82	5	–
Stab wound, *n* (%)	189	184	5	–
Renal trauma, *n* (%)	48 (1.5)	33 (1.1)	15 (8.5)	< 0.001
Trauma brain injury, *n* (%)	950 (31)	879 (30)	71 (40)	0.006
TRISS	0.98 [0.90–0.99]	0.98 [0.92–0.99]	0.84 [0.38–0.96]	< 0.001
Prehospital characteristics				
Delay between trauma and hospital admission, min	77 [55–110]	75 [55–106]	90 [65–120]	< 0.001
GCS	15 (12–15)	15 (13–15)	14 (5–15)	< 0.001
Minimum SAP, mmHg	115 [100–130]	116 [100–130]	87 [70–111]	< 0.001
Minimum DAP, mmHg	70 [58–80]	70 [59–80]	50 [40–69]	< 0.001
Mean AP, mmHg	84 [70–95]	85 [73–95]	62 [52–82]	< 0.001
Maximum HR, bpm	93 [80–110]	91 [80–109]	110 [90–130]	< 0.001
Minimum SpO_2_, %	100 [98–100]	98 [96–100]	95 [88–98]	< 0.001
Use of vasopressors, *n* (%)	369 (11.9)	304 (10.4)	65 (36.7)	< 0.001
Hospital admission				
SAP, mmHg	124 [108–139]	124 [109–139]	102 [78–132]	< 0.001
DAP, mmHg	72 [61–83]	72 [61–83]	59 [45–79]	< 0.001
pH	7.36 [7.30–7.40]	7.36 [7.31–7.40]	7.26 [7.08–7.33]	< 0.001
Lactate, mM	2 [1.2–3]	1.9 [1.1–3]	4.0 [2.6–8.0]	< 0.001
Hemoglobin, g.dL^−1^	13 [11.4–14.3]	13 [11.5–14.4]	10.4 [7.8–12.3]	< 0.001
Fibrinogen, g.L^− 1^	2.3 [1.8–2.7]	2.3 [1.9–2.8]	1.6 [0.9–2.2]	< 0.001
Mechanical ventilation at day 1, *n* (%)	1550 (49.8)	1397 (47.6)	153 (86.4)	< 0.001
Surgery day 1, *n* (%)	2370 (76.2)	2231 (76)	139 (78.5)	0.4
Interventional radiology procedure, *n* (%)	146 (4.7)	118 (4)	28 (15.8)	< 0.001
Transfusion				
Hemorrhagic shock, *n* (%)	355 (11.4)	266 (9.1)	89 (50.3)	< 0.001
RBC transfusion, U	0 [0–2]	0 [0–2]	5 [0–12]	< 0.001
FFP transfusion, U	0 [0–0]	0 [0–0]	3 [0–8]	< 0.001
Platelets transfusion, U	0 [0–0]	0 [0–0]	0 [0–2]	< 0.001
Outcomes during hospital stay				
ICU length of stay, days	4 [2–12]	4 [2–11]	10 [4–24]	< 0.001
Hospital length of stay, days	10 [4–23]	10 [4–22]	21 [4–38]	< 0.001
Predicted mortality, *n* (%)	367 (11.8)	310 (10.6)	57 (32.2)	–
Mortality, *n* (%)	335 (10.8)	268 (9.1)	67 (37.9)	< 0.001

The predicted mortality was calculated according to the Trauma and Injury Severity Score (TRISS). Patients with no AKI or AKI stage R were compared with patients with AKI stage I or F. All data are described as mean ± SD or median [Q1-Q3]

*AIS* abbreviated injury score, *AP* arterial pressure, *DAP* diastolic arterial pressure, *FFP* fresh-frozen plasma, *GCS* Glasgow Coma Scale, *HR* heart rate, *ICU* Intensive care unit, *ISS* injury severity score, *MVA* motor vehicle accident, *RBC* red blood cells, *SAP* systolic arterial pressure, *SAPS* Simplified Acute Physiology Score, *SD* standard deviation, *SOFA* sequential organ failure assessment score, *SpO*_*2*_ pulse oximeter oxygen saturation, *U* units of blood products

### Incidence of AKI

The prevalence of AKI in the overall population was 13% (CI 11.8–14.2) including 7% (CI 6.1–7.9) of patients with stage R, 3.7% (CI 3.0–4.4) of patients with stage I and 2.3% (CI 1.7–2.8) with stage F (Table 2). The incidence of AKI rose to 20.9%, 28.3% and 42.5% in the subgroups of trauma patients with an ISS > 16, in patients receiving at least one unit of packed RBC concentrate and in patients presenting with hemorrhagic shock, respectively (Table 2). AKI occurred early with a median delay of creatinine peak not exceeding 2 days (Table 3): 96% of patients with AKI (*n* = 388) had early AKI, as they reached the creatinine peak within 5 days after trauma, while 4% of patients with AKI (*n* = 17) had late AKI (8 patients with AKI stage R and 9 patients with AKI stage F) and reached the creatinine peak after 5 days. Renal replacement therapy was used in 49 patients (1.6%).

**Table 2:** Prevalence of AKI in the overall population and in three subgroups of patients

Group	No AKI	RIFLE R	RIFLE I	RIFLE F
Overall population (*n* = 3111), *n* (%)	2706 (87.0)	219 (7.0)	116 (3.7)	70 (2.3)
ISS ≥ 16 (*n* = 1549), *n* (%)	1225 (79.1)	161 (10.4)	102 (6.6)	61 (3.9)
≥ 1 unit of packed RBC during ICU stay (*n* = 877), *n* (%)	629 (71.7)	117 (13.3)	76 (8.7)	55 (6.3)
Hemorrhagic shock (*n* = 355), *n* (%)	204 (57.5)	59 (16.6)	49 (13.8)	43 (12.1)

*AKI* acute kidney injury, *ICU* Intensive Care Unit, *ISS* Injury Severity Score, *RBC* red blood cells, *RIFLE* risk, injury, failure, loss of function and end-stage renal disease

**Table 3:** Creatinine peak value, median time to creatinine peak and use of renal replacement therapy (RRT) according to acute kidney injury severity

	RIFLE R	RIFLE I	RIFLE F
	*n* = 219	*n* = 116	*n* = 70
Creatinine peak, μmol.L^−1^	107 ± 34	166 ± 68	256 ± 126
Median time to creatinine peak, days	0 [0–1]	1 [0–1]	2 [1–3]
RRT, *n* (%)	2 (0.9)	12 (10.3)	35 (50)

R, I and F are the risk, injury and failure stages in the risk, injury, failure, loss of function and end-stage renal disease (RIFLE) classification

### Characteristics of trauma patients with AKI

Patients with early AKI were more severely ill than patients with no AKI or stage-R AKI, as reflected by worse hemodynamic variables, more severe TBI (initial GCS) and more blood product transfusion (Table 1, Additional file 3). Among variables associated with severe AKI in the univariate analysis, collinear variables were TBI, initial GCS, and minimum prehospital MAP and SAP. Initial GCS and minimum MAP were retained for the final analysis. There were 2345 patients included in the multivariate model (missing values led us to exclude 766 patients whose characteristics are reported in Additional file 2). The following factors were associated with AKI stage I or F (Table 4): presence of hemorrhagic shock, blood lactate, minimum prehospital MAP, maximum prehospital heart rate, the ISS and secondary transfer to a trauma center. The AUC-ROC of the model was 0.85 (0.82–0.88). The same predictors were selected in the model predicting AKI all stages (R, I or F) except maximum prehospital heart rate that was not retained in the final model and renal trauma that was included in the final model (Additional file 3). The AUC-ROC of the latter model was 0.80 (0.77–0.83). There was no significant interaction.

**Table 4:** Risk factors associated with the occurrence of early AKI stage I or F in a stepwise logistic regression model

Parameter	OR	CI 95%	*p* value
ISS	1.035	1.021–1.049	< 0.001
Hemorrhagic shock	2.774	1.572–4.895	< 0.001
Lactate	1.093	1.022–1.170	0.004
Maximal prehospital HR	1.008	1.001–1.015	0.019
Minimum prehospital MAP	0.988	0.978–0.998	0.026
Direct transfer to trauma center	0.499	0.276–0.898	0.032
Renal trauma	2.303	0.834–6.361	0.073
Age	1.008	0.997–1.018	0.16
Angio-embolization	1.554	0.839–2.881	0.153
Blunt/penetrating trauma	0.584	0.238–1.429	0.193
Prehospital vasopressor use	0.897	0.500–1.607	0.739
Minimum prehospital SpO_2_	1.001	0.986–1.015	0.968
Fibrinogen	1.071	0.823–1.393	0.586
Initial GCS	0.998	0.952–1.047	0.939

Missing values (among which lactate value accounted for 80% of cases) led us to analyze 2345 patients in the model. Characteristics of the 766 patients excluded from the analysis are presented in Additional file 3. Results are given as odds ratio (OR) and 95% confidence interval (CI). The Lemeshow test was used (*p* = 0.10). The AUC of the model = 0.854 (0.820–0.881)

*MAP* mean arterial pressure, *GCS* Glasgow Coma Scale, *HR* heart rate, *ISS* injury severity score, *SpO*_*2*_ pulse oximeter oxygen saturation Hosmer Lemeshow Test (*p* = 0.10)

### Rhabdomyolysis and AKI

CK peak was available in 1382 patients (missing values led us to exclude 963 patients whose characteristics are reported in Additional file 2). The CK peak value reached 1052 U/L (403–2897) in patients with no AKI or R-stage AKI while it reached 3942 U/L (1481–11,338) in patients with AKI stage I or F (*p* < 0.0001). The CK peak value reached 977 U/L (383–2417) in patients with no AKI while it reached 2800 U/L (1032–7938) in patients with AKI stage R, I or F (*p* < 0.0001). When adding CK peak to the factors previously included in the multivariate model, it was significantly associated with AKI stage I or F (OR 1.041 (1.015–1.069) per step of 1000 U/mL, *p* < 0.001) and with AKI of all stages (OR 1.041 (1.010–1.073) per step of 1000 U/mL, *p* < 0.001) (Additional file 4).

### Prediction of AKI

**Fig. 1:**
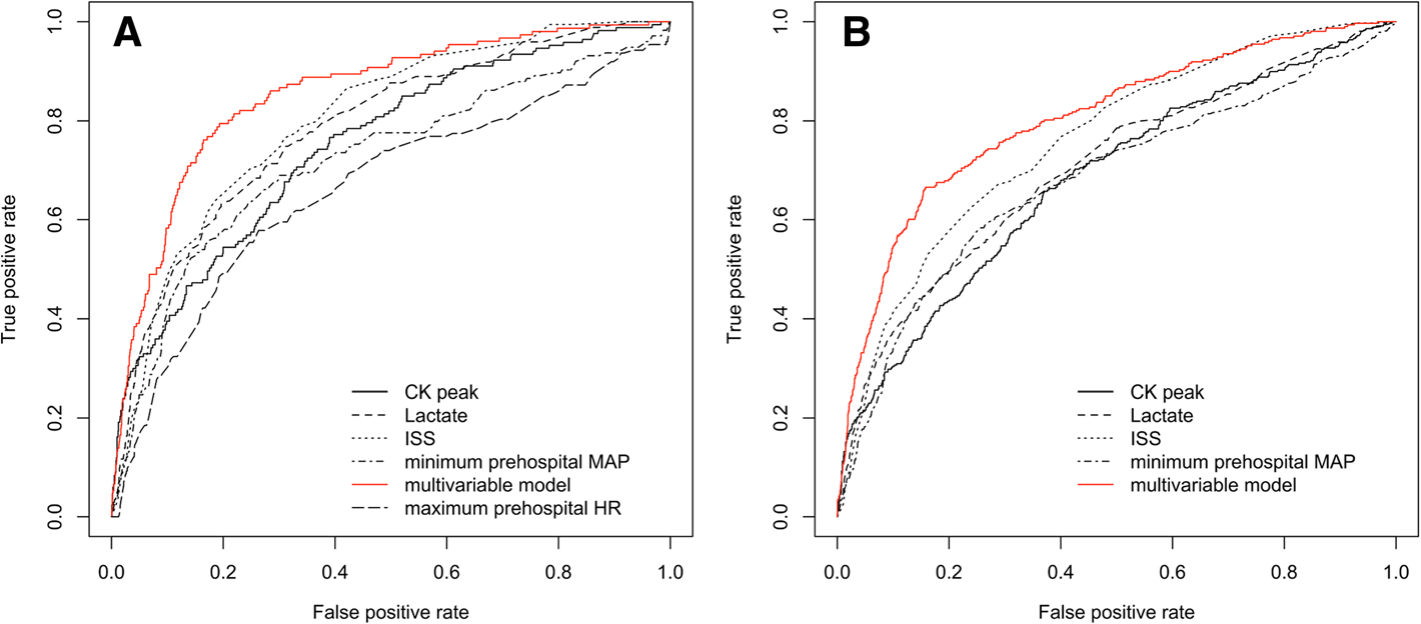
**a** Receiver operating characteristics (ROC) curves for prediction of acute kidney injury (AKI) (stage I or F) with Injury Severity Score (ISS) (AUC = 0.79 (0.75–0.83)), blood lactate (AUC = 0.77 (0.73–0.81)), Creatine kinase (CK) peak (AUC = 0.73 (0.69–0.78)), minimum prehospital mean arterial pressure (MAP) (AUC = 0.70 (0.65–0.75)) and maximum prehospital heart rate (HR) (AUC = 0.66 (0.61–0.71)). The multivariate model includes the following variables: presence of hemorrhagic shock, blood lactate, minimum prehospital MAP, maximum prehospital heart rate, ISS and secondary transfer to a trauma center (Table 4). The AUC-ROC of the multivariate model is 0.85 (0.82–0.88). **b** ROC curves for prediction of AKI (stage R, I or F) with ISS (AUC = 0.76 (0.73–0.78)), blood lactate (AUC = 0.70 (0.67–0.73)), CK peak (AUC = 0.68 (0.65–0.71)), minimum prehospital MAP (AUC = 0.68 (0.65–0.71)). The multivariate model includes the following variables: presence of hemorrhagic shock, blood lactate, minimum prehospital MAP, ISS, secondary transfer to a trauma center and presence of severe renal trauma (Additional file 3). The AUC-ROC of the multivariate model is 0.80 (0.78–0.83)

We assessed the discriminating power of each parameter independently associated with AKI. The ISS, blood lactate, CK peak, minimum prehospital MAP and maximum prehospital heart rate had an AUC-ROC of 0.80 (0.77–0.83), 0.79 (0.75–0.82), 0.75 (0.71–0.79), 0.72 (0.68–0.77) and 0.66 (0.62–0.71), respectively, to predict AKI stage I or F (Fig. 1a). The AUC-ROC for prediction of AKI of all stages was respectively 0.76 (0.73–0.78), 0.70 (0.67–0.73), 0.68 (0.65–0.71) and 0.68 (0.65–0.71) for ISS, blood lactate, CK peak and minimum prehospital MAP (Fig. 1b). Additional analysis, including sensitivity, specificity, PPV, NPV, PLR, NLR and optimal cutoff is available in Additional file 5.

### AKI and outcome after trauma

In a stepwise logistic regression model, AKI was independently associated with a twofold increase in ICU mortality (OR = 2.321 (1.389–3.85) for AKI stage I or F (*p* = 0.001) and OR = 1.943 (1.300–2.890) for AKI stage R, I or F (*p* = 0.001)) (Additional file 6).

## Discussion

In this 3-year multicenter observational study, we found that AKI occurred in 13% of trauma patients but this increased up to 42% in patients presenting with hemorrhagic shock. Second, we also found that AKI occurred early, with 96% of AKI diagnosed within the first 5 days after traumatic injury. Third, the model predicting AKI performed well and provided early risk factors for AKI that are markers of hypoperfusion and metabolic aggression (admission lactate value, hemorrhagic shock, minimum prehospital MAP and maximal prehospital heart rate), injury severity (ISS), renal trauma and delayed admission. Fourth, rhabdomyolysis severity (CK peak) was an additional independent risk factor for AKI. Fifth, AKI was independently associated with an increased risk of ICU mortality. To our knowledge, this is the largest multicenter cohort of trauma patients in which AKI risk factors were assessed. For the first time, in association with hospital variables collected early on admission, we report prehospital variables to predict the occurrence of AKI after trauma. This is all the more relevant that AKI has an early onset after trauma, thereby calling for its early prediction to direct treatment aiming to prevent AKI.

The overall incidence of 13% of AKI is consistent with previous studies, though wide ranges of AKI proportions from 1 to 50% have been reported in trauma patients [2–6, 8, 9, 17, 18, 28, 29]. These large differences between studies are likely to be due to AKI criteria, length of follow up and severity of trauma that are different from one study to another [11]. Our cohort of patients had a median ISS of 14, which is less severe than reported elsewhere [2, 8]. However, 13% of AKI is significant and calls for attention to patients’ kidney function after trauma. Moreover, when considering subpopulations of patients with an ISS > 16 or requiring blood transfusion, AKI prevalence rose to 21% and 28%, respectively, which was similar to studies including severely injured patients admitted to ICU [6, 9, 17]. The percentage of AKI (42%) in patients presenting with hemorrhagic shock is among the highest reported in the literature [2, 4]. In the future, studies investigating AKI prevention should take into account this highly variable rate according to the trauma population to which they refer. Our large proportion of patients with early AKI is consistent with recent findings showing that post-injury multiorgan failure develops early in the course of post-trauma care (median delay of 2 days) [30, 31], without bimodal distribution [32].

The severity of trauma injuries (ISS) was associated with AKI in our study, which is inconsistently reported in other studies in the field of trauma [4, 5, 17]. A high ISS is not in itself an indicator of renal aggression but rather a marker of the amount of wounded tissue that may ultimately promote systemic inflammation and subsequently renal failure [33, 34]. Severe renal trauma (AIS ≥ 3) was also associated with the occurrence of AKI all stages, suggesting that severe renal parenchyma (or vessel) injuries can decrease functional nephron mass and subsequently lead to a decrease in glomerular filtration rate. We considered several hemodynamic variables in our model. Hemorrhagic shock (> 4 RBC units within 6 h), blood lactate, minimum prehospital diastolic arterial pressure (DAP) and maximum prehospital heart rate were associated with AKI. Blood transfusion is a marker of the amount of bleeding, which in and of itself can lead to hypoperfusion and AKI [4, 6, 35]. Blood lactate is reported elsewhere to be associated with AKI [7, 17]. Its value on arrival to the hospital indicates, even in the absence of hypotension, the importance of cumulated metabolic debts due to tissue hypoperfusion [36]. Interestingly, low prehospital MAP, an early available parameter, added independent information to hemorrhagic shock and blood lactate to predict AKI.

Patients directly transported to a trauma center were less likely to experience AKI than those who were secondarily admitted to the referral trauma center. The most common reasons for such transfers include injuries requiring specialized care that cannot be provided in the first hospital the patient was admitted to, thereby corresponding to under-triage. Early admission to the referral trauma center likely allows earlier hemorrhage control and injury care, preventing renal aggression. Surprisingly, age was not significantly associated with AKI. This has been reported elsewhere [4, 17] and may be explained by the youthful cohort in our study. We report no significant association between use of vasopressors and AKI. No previous study has ever addressed the link between AKI and the use of vasopressors in trauma patients. However, previous retrospective studies have highlighted an association between the use of vasopressors and mortality in trauma patients [37–39]. By correcting for confounding factors, our group recently reported in a large multicenter study that early use of vasopressors was not associated with mortality in trauma patients presenting with hemorrhagic shock [15]. Similar to this study, our predictive model takes into account the most relevant factors of illness severity, which may explain the lack of association between vasopressors and AKI. Severity of rhabdomyolysis as assessed by the CK peak was a risk factor for AKI. CK is not nephrotoxic in itself but its level is a measure of the severity of intramuscular content release and as such, the higher the CK peak the more intense the release of intramuscular mediators that have nephrotoxic properties. However, CK peak is reached with a median time of 17 h after trauma and cannot be a relevant early predictor of AKI [40]. Future studies should focus on early prediction of severe rhabdomyolysis to indicate therapy aiming to prevent rhabdomyolysis-induced AKI. Regarding exposure to nephrotoxic agents, the use of angio-embolization was not a significant risk factor for AKI in the predictive models. This might be due to the negligible nephrotoxic risk promoted by the use of contrast agents for angio-embolization in comparison with inflammation, hypotension and tissue hypoperfusion. However, we cannot rule out contrast nephrotoxicity since every patient underwent contrast-enhanced (total body) CT, which may have increased baseline risk of AKI in the whole population. Moreover, we could not report the amount of contrast agent used during angio-embolization procedures.

Regarding AKI prediction, none of the variables associated with AKI performed satisfactorily to individually discriminate the occurrence of AKI stage I or F. However, by using prehospital and early hospital variables, our logistic regression model performed well to predict AKI stage I or F with an AUC-ROC of 0.85. The model for AKI all stages (R, I or F) performed worse, with an AUC-ROC of 0.80. By comparison, in a well-conducted study, Haines et al. recently used hospital variables to build a model predicting stage 2 or 3 AKI (Kidney Disease Improving Global Outcome (KDIGO) classification) in trauma patients, with an AUC of 0.81, while their model predicting AKI of all stages had an AUC of 0.77 [35]. Taking into account additional AKI risk factors such as AKI biomarkers or plasma inflammatory markers might help to improve AKI prediction in future studies.

Several investigations have emphasized that AKI is independently linked to adverse clinical outcome in ICU patients [41] or in trauma patients [3–5, 17, 42]. In the present study, AKI was independently associated with mortality even taking into account standard scores for trauma severity like TRISS. Thus, renal failure represents an additional marker of high risk of mortality in trauma patients.

There are several limitations to our study. This is a retrospective study, though data used in the study were prospectively collected and registered in a research database. Since blood lactate and CK were not systematically measured in trauma patients during the study period, missing values led us to exclude patients from the predictive models. However, the number of AKI patients in our sample far exceeded the number of events that is recommended (at least 10 events (10 patients with AKI) per candidate variable) when building a predictive model and therefore is expected to provide robust estimates [27]. Data on comorbidities were not collected in our database, including data on chronic kidney disease, which is regularly reported to be a risk factor for AKI in the ICU. However, our trauma patients are young and are likely to have no past medical history, in contrast to the general ICU population. Reporting data on the use of nephrotoxic agents (synthetic colloids, antibiotics and regular angiotensin converting enzyme inhibitors) would have added relevant information to the study but these data are not collected in our database. The TraumaBase group used RIFLE criteria to categorize AKI at the time of the study, which may have underestimated AKI in comparison with more recent classifications (i.e. KDIGO) that are more sensitive for AKI diagnosis in ICU patients [43]. AKI diagnosis was only based on creatinine criteria. By not taking into account urine output criteria, we may have further underestimated AKI prevalence. We used the lowest serum creatinine value during the first 5 days of the ICU stay as baseline creatinine. This may have resulted in a lower estimate of baseline creatinine than back-calculation through the MDRD formula with a glomerular filtration rate of 75 mL/min per 1.73 m^2^. Nevertheless, the definition we used was reported to be more accurate to diagnose AKI in ICU patients than creatinine estimated by MDRD [44], especially in trauma patients whose baseline serum creatinine is overestimated by MDRD [20]. Last, this study demonstrated an association between risk factors and AKI but cannot establish causality.

## Conclusion

AKI has an early onset and is independently associated with mortality in trauma patients. Its prevalence varies by a factor of 3 according to the severity of injuries and hemorrhage. Prehospital and data on risk factors collected early after hospital admission can be effective in the early prediction of AKI after trauma. Hence, studies aiming to prevent AKI should target patients at high risk of AKI and investigate therapies early in the course of trauma care.

## Additional files

Additional file 1: French Vittel triage criteria. (DOCX 30 kb)

Additional file 2: General characteristics of patients with missing data. (DOCX 23 kb)

Additional file 3: Univariate analysis and stepwise logistic regression model including risk factors associated with the occurrence of AKI of all stages (R, I or F). (DOCX 32 kb)

Additional file 4: Risk factors associated with the occurrence of AKI stage I or F and with the occurrence of AKI of all stages (R, I or F) in a stepwise logistic regression model including CK peak. (DOCX 20 kb)

Additional file 5: Predictive performances of different models and variables for AKI stage I or F and for AKI of all stages (R, I or F). (DOCX 16 kb)

Additional file 6: Assessment of AKI stage I or F and AKI of all stages (R, I or F) as risk factors associated with mortality in a stepwise logistic regression model. (DOCX 29 kb)

## Abbreviations

AIS: Abbreviated Injury Scale
AKI: Acute kidney injury
AUC-ROC: Area under the curve of the receiver operating characteristic
CI: Confidence intervals
CK: Creatine kinase
CT: Computed tomography
DAP: Diastolic arterial pressure
FFP: Fresh-frozen plasma
GCS: Glasgow Coma Scale
HR: Heart rate
ICU: Intensive care unit
ISS: Injury Severity Score
MDRD: Modification of diet in renal disease
NLR: Negative likelihood ratio
NPV: Negative predictive value
OR: Odds ratio
PLR: Positive likelihood ratio
PPV: Positive predictive value
RBC: Red blood cells
RIFLE: Risk, injury, failure, loss of kidney function and end-stage kidney disease
ROC: Receiver operating characteristic
RRT: Renal replacement therapy
SAP: Systolic arterial pressure
SAPS II: Simplified Acute Physiology Score
SD: Standard deviation
SOFA: Sequential Organ Failure Assessment
SpO_2_: Pulse oximeter oxygen saturation
TBI: Traumatic brain injury
TRISS: Trauma-Related Injury Severity Score
