# Supplementary material for: Prevalence and risk factors for acute kidney injury among trauma patients: a multicenter cohort study
Source: Crit Care. 2018 Dec 18;22:344. doi: 10.1186/s13054-018-2265-9 (PMC6299611; doi:10.1186/s13054-018-2265-9)
Supplement: Supplementary file 1 — French Vittel triage criteria. (DOCX 30 kb) [file 13054_2018_2265_MOESM1_ESM.docx]

**Additional file 1**: The Vittel algorithm is applied from step 1 to 5. Presence of one criterium suggests to transport patients to a level one trauma center except step 5 for which a decision is made on a case by case basis. The Vittel criteria were introduced by the « Société Française de Médecine d’Urgence (SFMU) » in 2002. GCS = Glasgow Coma Scale, SpO_2_ = pulse oximeter oxygen saturation, SBP = Systolic blood pressure

Age > 65

Cardiac insufficiency, respiratory failure or ischemic heart disease

Pregnancy (2d, 3d trimester)

Coagulation disorders

Mechanical ventilation

Volume load > 1000 mL

Vasopressor

Shock trousers

Step five (medical history)

Step four (resuscitation)

Penetrating trauma of head, neck, thorax, abdomen

Flail chest

Severe burn

Pelvic fracture

Suspicion of medullar injury

Amputation at or above wrist or ankle level

Acute limb ischemia

Step three (anatomical injuries)

Step one (physiological signs)

GCS < 13 and/or

SBP < 90 mmHg and/or

SpO2 < 90%

Step two (global assessment of speed and mechanism)

Ejection from vehicle

Death in same passenger compartment

Fall > 6m

Victim thrown or projected

Global assessement of speed and potential injuries:

Vehicle deformation, estimated vehicle speed, helmet absent, seat belt not fastened

Blast
