# Supplementary material for: Prevalence and risk factors for acute kidney injury among trauma patients: a multicenter cohort study
Source: Crit Care. 2018 Dec 18;22:344. doi: 10.1186/s13054-018-2265-9 (PMC6299611; doi:10.1186/s13054-018-2265-9)
Supplement: Supplementary file 2 — General characteristics of patients with missing data. (DOCX 23 kb) [file 13054_2018_2265_MOESM2_ESM.docx]

| **Characteristics** | **Number of trauma patients admitted from May 2011 to July 2014**  **N=3488** | |  | **Patients suitable to assess AKI risk factors (excluding CK)**  **N=3111** | |  | **Patients suitable to assess AKI risk factors (including CK)**  **N=2345** | |
| --- | --- | --- | --- | --- | --- | --- | --- | --- |
|  | **Analysed for AKI prevalence** | **Missing values for AKI assessment** |  | **Analysed for AKI risk factors** | **Missing values** |  | **Analysed for AKI risk factors** | **Missing CK values** |
|  | **n=3111** | **n = 377** |  | **n=2345** | **n=766** |  | **n=1382** | **n=963** |
| **General characteristics** |  |  |  |  |  |  |  |  |
| Age, year | 38 ± 18 | 37 ± 17 |  | 38 ± 17 | 39 ± 17 |  | 38 ± 17 | 38 ± 17 |
| Ratio Female/Male, n(%) | 2428/683 (78/22) | 309/68 (82/18) |  | 1818/527 (78/22) | 610/156 (80/20) |  | 1068/314 (77/23) | 740/213 (78/22) |
| Direct transfer to trauma center, n(%) | 2589 (83.2) | 336 (89.1) |  | 2119 (90.4) | 465 (61) |  | 1240 (89.8) | 879 (91.3) |
| SAPS II | 21 [11- 38] | 21 [13-40] |  | 22 [12-40] | 16 [9-29] |  | 22 [12-39] | 22 [12-40] |
| SOFA 24h | 2 [0-6] | 2 [0-8] |  | 2 [0-7] | 1 [0-4] |  | 2 [0-7] | 2 [0-7] |
| ISS | 14 (9-25) | 16 [6-25] |  | 16 (9-25) | 13 [8-22] |  | 16 (9-25) | 16 (9-25) |
| Blunt /Penetrating, n(%) | 2835/276 (91/9) | 334/43 (89/11) |  | 2149/196 (92/8) | 686/80 (90/10) |  | 1252/130 (91/9) | 897/66 (93/7) |
| Trauma brain injury, n(%) | 950 (31) | 92 (24.4) |  | 726 (31) | 219 (28.7) |  | 450 (32.5) | 276 (28.7) |
| **Prehospital characteristics** |  |  |  |  |  |  |  |  |
| GCS | 15 (12-15) | 15 [11-15] |  | 15 [11-15] | 15 [14-15] |  | 15 [11-15] | 15 [12-15] |
| Minimum SAP, mmHg | 115 [100-130] | 115 [98-130] |  | 114 [97-130] | 120 [101-130] |  | 114 [97-130] | 113 [99-130] |
| Minimum DAP, mmHg | 70 [58-80] | 68 [54-80] |  | 69 [56-80] | 70 [60-80] |  | 69 [55-80] | 70 [58-80] |
| Maximum HR, bpm | 87 [75-102] | 90 [80-106] |  | 94 [80-110] | 90 [78-107] |  | 94 [80-110] | 93 [80-110] |
| Minimum SpO_2_, % | 100 [98-100] | 98 [95-100] |  | 98 [95-100] | 98 [96-100] |  | 98 [95-100] | 98 [95-100] |
| Use of vasopressors, n (%) | 369 (11.9) | 58 (15.4) |  | 306 (13.0) | 70 (9.2) |  | 177 (12.8) | 129 (13.4) |
| **Hospital admission** |  |  |  |  |  |  |  |  |
| SAP, mmHg | 124 [108-139] | 120 [107-136] |  | 123 [106-139] | 125 [111-140] |  | 124 [105-139] | 122 [107-138] |
| DAP, mmHg | 72 [61-83] | 70 [60-80] |  | 71 [60-83] | 72 [62-82] |  | 72 [60-83] | 70 [60-82] |
| Hemoglobine, g.dL^-1^ | 13 [11.4-14.3] | 13.2 [11.6-14.3] |  | 12.9 [11.3-14.2] | 13.2 [11.6-14.6] |  | 12.8 [11.2-14.2] | 13 [11.3-14.3] |
| Fibrinogen, g.L^-1^ | 2.3 [1.8-2.7] | 2.2 [1.8-2.6] |  | 2.3 [1.8-2.6] | 2.5 [2-3] |  | 2.2 [1.8-2.6] | 2.3 [1.9-2.7] |
| **Transfusion** |  |  |  |  |  |  |  |  |
| Hemorrhagic shock, n(%) | 355 (11.4) | 52 (13.8) |  | 303 (12.9) | 52 (6.8) |  | 173 (12.6) | 130 (13.4) |
| **Outcomes during hospital stay** |  |  |  |  |  |  |  |  |
| ICU length of stay, days | 4 [2-12] | 2 [1-4] |  | 5 [2-13] | 3 [2-9] |  | 5 [2-13] | 4 [2-13] |
| Hospital length of stay, days | 10 [4-23] | 7 [3-24] |  | 11 [4-25] | 9 [3-18] |  | 11 [4-25] | 11 [3-23] |
| Mortality, n(%) | 335 (10.8) | 40 (10.6) |  | 267 (11.4) | 68 (8.9) |  | 159 (11.5) | 108 (11.2) |

**Additional file 2** :General characteristics of patients with missing data

This additional file shows the characteristics of the patients included in the analysis and those excluded from the analysis to assess AKI prevalence, risk factors of AKI (excluding CK peak) and risk factors of AKI (including CK peak).

The first column shows the characteristics of the patients included in the analysis to assess AKI prevalence (n=3111) and those excluded from the analysis because of missing values regarding renal function (n=377). Excluded patients had a slightly higher ISS and proportion of prehospital vasopressor use than included patients. Hemodynamic parameters, coagulation test and mortality were similar.

The second column shows characteristics of the 2345 patients included in the analysis to assess risk factors for AKI (excluding CK peak) and those excluded from the analysis because of missing value (n=766 among which 80% missing lactate values and 20% other missing values). Excluded patients were less severe than the patients included in the multivariable model as attested by a slightly lower ISS, a lower mortality and a lower proportion of hemorrhagic shock and vasopressor use. Since blood lactate accounted for 80% of missing values in the 766 excluded patients, it is likely that blood lactate was not measured because this subgroup of patients was deemed less severe by the physician in charge on arrival to the trauma center.

The third column shows the characteristics of the 1382 patients included in the analysis to assess risk factors for AKI (including CK peak) and those excluded from the analysis because of missing CK values (n=963). The characteristics of the patients included in the analysis were similar to those of the patients excluded from the analysis.

CK = Creatine Kinase, DAP = diastolic arterial pressure, GCS = Glasgow Coma Scale, HR = Heart Rate, ICU = Intensive care unit, ISS = injury severity score, RBC = red blood cells, SAP = Systolic arterial pressure, SAPS = Simplified Acute Physiology Score, SOFA = sequential organ failure assessment score, SpO_2_ = pulse oximeter oxygen saturation
