# Supplementary material for: Prevalence and risk factors for acute kidney injury among trauma patients: a multicenter cohort study
Source: Crit Care. 2018 Dec 18;22:344. doi: 10.1186/s13054-018-2265-9 (PMC6299611; doi:10.1186/s13054-018-2265-9)
Supplement: Supplementary file 3 — Univariate analysis and stepwise logistic regression model including risk factors associated with the occurrence of AKI of all stages (R, I or F). (DOCX 32 kb) [file 13054_2018_2265_MOESM3_ESM.docx]

| **Characteristics** | **No early AKI n = 2723** | **Early AKI stage R, I or F  n = 388** | **p-value** |
| --- | --- | --- | --- |
|  |  |  |  |
| **General characteristics** |  |  |  |
| Age, year | 38.2 ± 17.5 | 38.9 ± 17.0 | 0.454 |
| Male sex, n(%) | 2122 (77.9) | 306 (78.8) | 0.596 |
| Direct transfer to trauma center, n(%) | 2278 (83.7) | 311 (80.0) | 0.081 |
| SAPS II | 18 [10 – 34] | 41 [26 – 60] | <0.001 |
| SOFA 24h | 1 [0 – 5] | 8 [4 – 12] | <0.001 |
| ISS | 13 [8 – 22] | 27 [17 – 38] | <0.001 |
| Blunt, n(%) | 2467 (90.6) | 368 (94.9) | 0.005 |
| Penetrating, n(%) | 256 (9 .4) | 20 (5.1) | 0.005 |
| Renal trauma, n(%) | 25 (0.9) | 23 (5.9) | <0.001 |
| Trauma brain injury, n(%) | 780 | 172 (44.4) | <0.001 |
| TRISS | 0.98 [0.93 – 0.99] | 0.89 [0.52 – 0.97] | <0.001 |
| Predicted Mortality, n(%) | 260 (9.6) | 107 (27.6) |  |
| **Prehospital characteristics** |  |  |  |
| Delay between trauma and hospital admission, min | 75 [54-105] | 90 [60-120] | <0.001 |
| GCS | 15 [13-15] | 14 [6-15] | <0.001 |
| Minimum SAP, mmHg | 117 [100-130] | 95 [76-120] | <0.001 |
| Minimum DAP, mmHg | 70 [60-80] | 57 [42-70] | <0.001 |
| Mean AP, mmHg | 86 [73-95] | 70 [55-87] | <0.001 |
| Maximum HR, bpm | 80 [91-108] | 104 [82-124] | <0.001 |
| Minimum SpO_2_, % | 98 [96-100] | 96 [90-99] | <0.001 |
| Use of vasopressors, n (%) | 243 (8.9) | 126 (32.4) | <0.001 |
| **Hospital admission** |  |  |  |
| SAP, mmHg | 125 [110-139] | 110 [85-135] | <0.001 |
| DAP, mmHg | 72 [62-83] | 64 [50-79] | <0.001 |
| pH | 7.36 [7.32-7.40] | 7.30 [7.18-7.36] | <0.001 |
| Lactate, mM | 1.9 [1.1-2.9] | 3.0 [2.0-6.0] | <0.001 |
| Hemoglobine, g.dL^-1^ | 13.1 [11.7-14.4] | 11.3 [8.8-13.0] | <0.001 |
| Fibrinogen, g.L^-1^ | 2.3 [2.0-2.8] | 1.8 [1.2-2.4] | <0.001 |
| Mechanical ventilation at day 1, n(%) | 1233 (45.3) | 317 (81.6) | <0.001 |
| Surgery day 1, n(%) | 2065 (75.8) | 305 (78.6) | 0.235 |
| Interventional radiology procedure, n(%) | 102 (3.7) | 44 (11.2) | <0.001 |
| **Transfusion** |  |  |  |
| Hemorrhagic shock, n(%) | 209 (7.7) | 146 (37.6) | <0.001 |
| RBC transfusion, U | 0 [0-0] | 3 [0-9] | <0.001 |
| FFP transfusion, U | 0 [0-0] | 0 [0-6] | <0.001 |
| Platelets transfusion, U | 0 [0-0] | 0 [0-1] | <0.001 |
| **Outcomes during hospital stay** |  |  |  |
| ICU length of stay, days | 4 [2-10] | 9 [4-21] | <0.001 |
| Hospital length of stay, days | 10 [4-21] | 18 [5-38] | <0.001 |
| Mortality, n(%) | 238 (8.7) | 117 (30.2) | <0.001 |

**Additional file 3 - a**: Univariate analysis of general, physiologic and injury severity characteristics of the patients without AKI or with AKI (all stages). The predicted mortality was calculated according to TRISS value. Comparisons were done between patients with no AKI or AKI stage R and patients with AKI stage I or F. All data are described as mean ±SD or median [Q1-Q3]. AIS = abbreviated injury score, AP = arterial pressure, DAP = diastolic arterial pressure, FFP = fresh frozen plasma, GCS = Glasgow Coma Scale, HR = Heart Rate, ICU = Intensive care unit, ISS = injury severity score, MVA = motor vehicle accident, RBC = red blood cells, SAP = Systolic arterial pressure, SAPS = Simplified Acute Physiology Score, SD=standard deviation, SOFA = sequential organ failure assessment score, SpO_2_ = pulse oximeter oxygen saturation, TRISS = Trauma and Injury Severity Score

| **Parameter** | **OR** | **CI 95%** | **p-value** |
| --- | --- | --- | --- |
| ISS | 1.033 | 1.022 – 1.044 | <0.001 |
| Hemorrhagic shock | 2.555 | 1.699 – 3.842 | <0.001 |
| Lactate | 1.105 | 1.040 – 1.174 | <0.001 |
| Renal trauma | 3.597 | 1.579 – 3.597 | 0.003 |
| Direct transfer to trauma center | 0.604 | 0.391 – 0.933 | 0.026 |
| Minimum prehospital MAP | 0.991 | 0.984 – 0.999 | 0.035 |
| Blunt/penetrating trauma | 0.539 | 0.289 – 1.003 | 0.06 |
| Maximal prehospital HR | 1.004 | 0.999 – 1.009 | 0.103 |
| Age | 1.005 | 1.013 – 0.997 | 0.180 |
| Angio-embolization | 0.959 | 0.556 – 1.652 | 0.869 |
| Prehospital vasopressor use | 1.118 | 0.732 – 1.707 | 0.615 |
| Minimum prehospital SpO_2_ | 1.002 | 0.991 – 1.014 | 0.731 |
| Fibrinogen | 1.030 | 0.841 – 1.262 | 0.807 |
| Initial GCS | 0.980 | 0.945 – 1.016 | 0.309 |

**Additional file 3 – b**: Risk factors associated with the occurrence of early AKI all stage (R, I or F) in a stepwise logistic regression model. Missing values (among which lactate value accounted for 80% of cases) led us to analyze 2345 patients in the model. Characteristics of the 766 patients excluded of the analysis are presented in additional file 3. Results are given as Odds Ratio (OR) and 95 % confidence interval (CI). MAP = mean arterial pressure, GCS = Glasgow coma scale, HR = heart rate, ISS = injury severity score, SpO_2_ = pulse oximeter oxygen saturation. Hosmer Lemeshow Test (p=0.13). AUC of the model = 0.802 (0.778-0.832).
