# Supplementary material for: Prevalence and risk factors for acute kidney injury among trauma patients: a multicenter cohort study
Source: Crit Care. 2018 Dec 18;22:344. doi: 10.1186/s13054-018-2265-9 (PMC6299611; doi:10.1186/s13054-018-2265-9)
Supplement: Supplementary file 4 — Risk factors associated with the occurrence of AKI stage I or F and with the occurrence of AKI of all stages (R, I or F) in a stepwise logistic regression model including CK peak. (DOCX 20 kb) [file 13054_2018_2265_MOESM4_ESM.docx]

| **Variable** | **OR** | **CI 95%** | **p-value** |
| --- | --- | --- | --- |
| ISS | 1.038 | 1.023 – 1.054 | <0.001 |
| CK peak (per 1000 U/L) | 1.043 | 1.016 – 1.071 | <0.001 |
| Lactate | 1.162 | 1.063 – 1.271 | <0.001 |
| Prehospital minimum MAP | 0.987 | 0.976 – 0.999 | 0.039 |
| Angio-embolization | 1.048 | 0.493 – 2.227 | 0.115 |
| Direct transfer to trauma center | 0.486 | 0.255 – 0.927 | 0.013 |
| Hemorrhagic shock | 1.885 | 1.022 – 3.477 | 0.042 |
| Prehospital maximum HR | 1.009 | 1.001 - 1.018 | 0.024 |
| Renal trauma | 2.568 | 0.907 – 7.273 | 0.057 |
| Age | 1.005 | 0.993 – 1.018 | 0.335 |
| Blunt/penetrating trauma | 0.456 | 0.135 – 1.541 | 0.210 |
| Prehospital vasopressor use | 0.758 | 0.406 – 1.414 | 0.432 |
| Prehospital minimum SpO_2_ | 0.993 | 0.975 – 1.011 | 0.356 |
| Fibrinogen | 1.068 | 0.754 – 1.511 | 0.711 |
| Initial GCS score | 1.003 | 0.947 – 1.062 | 0.891 |

**Additional file 4 - a**: Risk factors associated with the occurrence of AKI stage I or F in a stepwise logistic regression model including CK peak. Odds Ratio (OR) and 95 % confidence interval (CI). CK=Creatine Kinase, MAP =mean arterial pressure, GCS = Glasgow coma scale, HR = heart rate, ISS = injury severity score, SpO_2_ = pulse oximeter oxygen saturation. N=1382 patients. Characteristics of the 963 patients excluded of the analysis are presented in additional file 1. Hosmer Lemeshow Test (p=0.60). AUC of the model = 0.859 (0.830-0.897).

| **Variable** | **OR** | **CI 95%** | **p-value** |
| --- | --- | --- | --- |
| ISS | 1.037 | 1.023 – 1.051 | <0.001 |
| CK peak (per 1000 U/L) | 1.041 | 1.010 – 1.073 | <0.001 |
| Lactate | 1.188 | 1.103 – 1.279 | <0.001 |
| Hemorrhagic shock | 1.862 | 1.196 – 2.900 | 0.008 |
| Direct transfer to trauma center | 0.592 | 0.364 – 0.962 | 0.028 |
| Renal trauma | 2.646 | 1.040 – 6.730 | 0.038 |
| Prehospital minimum MAP | 0.991 | 0.983 – 0.999 | 0.045 |
| Angio-embolization | 0.660 | 0.344 – 1.266 | 0.167 |
| Prehospital maximum HR | 1.004 | 0.998 – 1.010 | 0.200 |
| Age | 1.001 | 1.000 – 1.002 | 0.521 |
| Blunt/penetrating trauma | 0.407 | 0.163 – 1.013 | 0.060 |
| Prehospital vasopressor use | 1.099 | 0.689 – 1.754 | 0.674 |
| Prehospital minimum SpO_2_ | 0.995 | 0.981 – 1.009 | 0.450 |
| Fibrinogen | 0.959 | 0.758 – 1.215 | 0.772 |
| Initial GCS score | 0.983 | 0.944 – 1.024 | 0.389 |

**Additional file 4 - b**: Risk factors associated with the occurrence of AKI stage R, I or F in a stepwise logistic regression model including CK peak. Odds Ratio (OR) and 95 % confidence interval (CI). CK=Creatine Kinase, MAP =mean arterial pressure, GCS = Glasgow coma scale, HR = heart rate, ISS = injury severity score, SpO_2_ = pulse oximeter oxygen saturation. N=1382 patients. Characteristics of the 963 patients excluded of the analysis are presented in additional file 1. Hosmer Lemeshow Test (p=0.25). AUC of the model = 0.802(0.777-0.839).
