# Supplementary material for: Prevalence and risk factors for acute kidney injury among trauma patients: a multicenter cohort study
Source: Crit Care. 2018 Dec 18;22:344. doi: 10.1186/s13054-018-2265-9 (PMC6299611; doi:10.1186/s13054-018-2265-9)
Supplement: Supplementary file 5 — Predictive performances of different models and variables for AKI stage I or F and for AKI of all stages (R, I or F). (DOCX 16 kb) [file 13054_2018_2265_MOESM5_ESM.docx]

| **Variable** | **Cut-off value** | **Sensitivity** | **Specificity** | **PPV** | **NPV** | **NLR** | **PLR** |
| --- | --- | --- | --- | --- | --- | --- | --- |
| ISS | 21 | 77 (70-82) | 69 (67-71) | 13 (11-15) | 98 (97-98) | 0.34 (0.26-0.44) | 2.5 (2.2-2.7) |
| CK peak | 1469 U/L | 77 (70-82) | 61 (59-63) | 17 (14-20) | 96 (95-97) | 0.38 (0.29-0.51) | 2.0 (1.8-2.2) |
| Minimum prehospital MAP | 76 mmHg | 69 (62-75) | 70 (68-72) | 13 (11-15) | 97 (96-98) | 0.44 (0.36-0.55) | 2.3 (2.0-2.6) |
| Lactate | 2.6 mmol/L | 75 (68-80) | 69 (67-71) | 14 (12-16) | 98 (97-98) | 0.36 (0.28-0.47) | 2.4 (2.2-2.7) |
| Maximum prehospital HR | 108/min | 58 (50-65) | 74 (72-75) | 12 (10-15) | 97 (96-97) | 0.57 (0.48-0.68) | 2.2 (1.9-2.5) |
| Multivariable model without CK | 0.06 | 79 (72-85) | 81 (79-82) | 22 (19-26) | 98 (98-99) | 0.25 (0.19-0.35) | 4.1 (3.7-4.6) |
| Multivariable model with CK | 0.11 | 76 (68-82) | 84 (82-86) | 35 (30-40) | 97 (96-98) | 0.29 (0.22-0.39) | 4.8 (4.1-5.6) |

**Additional file 5 - a**: predictive performances of different models and variables for AKI stage I or F prediction. 95% confidence intervals are given for each parameter. NLR = negative likelihood ratio, NPV = negative predictive value, PLR = positive likelihood ratio, PPV = positive predictive value of parameters. CK=Creatine Kinase, MAP=Mean Arterial Pressure, HR=Heart Rate, ISS=Injury Severity Score.

| **Variable** | **Cut-off value** | **Sensitivity** | **Specificity** | **PPV** | **NPV** | **NLR** | **PLR** |
| --- | --- | --- | --- | --- | --- | --- | --- |
| ISS | 24 | 63 (58-67) | 76 (74-77) | 27 (24-30) | 93 (92-94) | 0.349 (0.43-0.56) | 2.6 (2.3-2.9) |
| CK peak | 1455 U/L | 66 (61-70) | 63 (60-65) | 31 (28-34) | 88 (86-90) | 0.54 (0.47-0.63) | 1.8 (1.6-2.0) |
| Minimum prehospital MAP | 73 mmHg | 58 (53-63) | 75 (73-76) | 25 (22-28) | 93 (91-94) | 0.56 (0.49-0.63) | 2.3 (2.1-2.6) |
| Lactate | 2.3 mmol/L | 67 (62-71) | 64 (62-66) | 22 (20-25) | 93 (91-94) | 0.52 (0.45-0.60) | 1.8 (1.7-2.0) |
| Multivariable model without CK | 0.14 | 67 (61-72) | 84 (83-86) | 39 (35-43) | 94 (93-95) | 0.40 (0.34-0.46) | 4.2 (3.7-4.8) |
| Multivariable model with CK | 0.24 | 64 (58-70) | 85 (83-87) | 53 (47-58) | 90 (88-92) | 0.42 (0.36-0.49) | 4.4 (3.7-5.2) |

**Additional file 5 - b**: predictive performances of different models and variables for AKI stage R, I or F prediction. 95% confidence intervals are given for each parameter. NLR = negative likelihood ratio, NPV = negative predictive value, PLR = positive likelihood ratio, PPV = positive predictive value of parameters. CK=Creatine Kinase, MAP=Mean Arterial Pressure, HR=Heart Rate, ISS=Injury Severity Score.
