# Supplementary material for: Prevalence and risk factors for acute kidney injury among trauma patients: a multicenter cohort study
Source: Crit Care. 2018 Dec 18;22:344. doi: 10.1186/s13054-018-2265-9 (PMC6299611; doi:10.1186/s13054-018-2265-9)
Supplement: Supplementary file 6 — Assessment of AKI stage I or F and AKI of all stages (R, I or F) as risk factors associated with mortality in a stepwise logistic regression model. (DOCX 29 kb) [file 13054_2018_2265_MOESM6_ESM.docx]

| **Characteristics** | **Whole cohort n = 3111** | **Survivors**  **n = 2776** | **Non-survivors**  **n = 335** | **p-value** |
| --- | --- | --- | --- | --- |
|  |  |  |  |  |
| **General characteristics** |  |  |  |  |
| Age, year | 38 ± 18 | 38 ± 18 | 38 ± 17 | 0.71 |
| Male sex, n(%) | 2428 (78.0) | 2168 (78.1) | 260 (77.6) | 0.94 |
| Direct transfer to trauma center, n(%) | 2589 (83.2) | 2299 (82.8) | 290 (86.6) | 0.08 |
| Blunt, n(%) | 2835 (91.1) | 2516 (90.6) | 319 (95.2) | 0.005 |
| SAPS II | 21 [11- 38] | 18 [10- 32] | 63 [47-78] | <0.001 |
| SOFA 24h | 2 [0-6] | 0 [1-5] | 10 [8-13] | <0.001 |
| ISS | 14 (9-25) | 13 [8-22] | 33 [25-43] | <0.001 |
| Trauma brain injury, n(%) | 950 (31) | 715 (25.8) | 235 (70.1) | <0.001 |
| TRISS | 0.98 [0.90-0.99] | 0.98 [0.94-0.99] | 0.58 [0.15-0.87] | <0.001 |
| GCS | 15 [12-15] | 15 [14-15] | 5 [3-10] | <0.001 |
| Minimum prehosp SAP, mmHg | 115 [100-130] | 117 [100-130] | 90 [60-120] | <0.001 |
| Maximum prehosp HR, bpm | 93 [80-110] | 92 [80-109] | 99 [79-120] | 0.02 |
| Minimum prehosp SpO_2_, % | 100 [98-100] | 98 [96-100] | 99 [90-100] | <0.001 |
| Prehosp use of vasopressors, n (%) | 369 (11.9) | 208 (75.0) | 161 (48.1) | <0.001 |
| Lactate, mM | 2 [1.2-3] | 1.9 [1.1-2.9] | 2.1 [3.6-8.9] | <0.001 |
| Fibrinogen, g.L^-1^ | 2.3 [1.8-2.7] | 2.3 [2.0-2.8] | 1.6 [0.7-2.4] | <0.001 |
| Mechanical ventilation day 1, n(%) | 1550 (49.8) | 1229 (44.3) | 310 (92.5) | <0.001 |
| Hemorrhagic shock, n(%) | 355 (11.4) | 236 (8.5) | 119 (35.5) | <0.001 |
| RIFLE I or F, n(%) | 186 (6.0) | 115 (4.1) | 71 (21.2) | <0.001 |
| RIFLE R, I or F, n(%) | 405 (13.0) | 281 (10.1) | 123 (36.7) | <0.001 |

**Additional file 6 - a**: Univariate analysis of risk factors associated with mortality. GCS = Glasgow Coma Scale, HR = Heart Rate, ISS = injury severity score, prehosp=prehospital, RIFLE = Risk, Injury, Failure, Loss of function, End stage disease, SAP = Systolic arterial pressure, SAPS = Simplified Acute Physiology Score, SOFA = sequential organ failure assessment score, SpO_2_ = pulse oximeter oxygen saturation, TRISS = Trauma and Injury Severity Score.

We included in a stepwise logistic regression model the following variables:

-Minimum prehospital SAP

-Trauma brain injury

-GCS

-Hemorrhagic shock

-Direct transfer trauma center

-Blunt/penetrating

-Lactates

-Minimum prehospital SpO_2_

-Fibrinogen

-Prehospital use of vasopressor

-AKI RIFLE I or F (Table 4-b below)

-AKI RIFLE R, I or F (Table 4-c below)

-Mechanical Ventilation day 1

-ISS

TRISS, SOFA and SAPSII were not included to avoid redundancies since they integrate most of the abovementioned physiological variables

| **Variable** | **OR** | **CI 95%** | **p-value** |
| --- | --- | --- | --- |
| Trauma brain injury | 2.933 | 1.959 – 4.431 | <0.001 |
| GCS | 0.805 | 0.772 – 0.837 | <0.001 |
| Lactates | 1.241 | 1.171 – 1.317 | <0.001 |
| ISS | 1.039 | 1.026 – 1.052 | <0.001 |
| RIFLE I or F | 2.321 | 1.389 – 3.856 | 0.001 |
| Minimum prehosp SpO_2_ | 0.988 | 0.977 – 0.999 | 0.04 |

**Additional file 6 – b:** Risk factors associated with death. Among the 13 variables included in the model, only Trauma brain injury, GCS, Lactates, ISS, Minimum SpO_2_ and RIFLE I or F were independently associated with mortality. Hosmer and Lemeshow Test (p=0.25). AUC of the model = 0.93 (0.91-0.94). The analysis was conducted on 2362 patients. CI = Confidence Interval, OR = Odds ratio, prehosp=prehospital.

| **Variable** | **OR** | **CI 95%** | **p-value** |
| --- | --- | --- | --- |
| Trauma brain injury | 2.670 | 1.768 – 4.071 | <0.001 |
| GCS | 0.817 | 0.782 – 0.853 | <0.001 |
| Lactates | 1.247 | 1.168 – 1.333 | <0.001 |
| ISS | 1.038 | 1.025 – 1.052 | <0.001 |
| RIFLE R, I or F | 1.943 | 1.300 – 2.890 | 0.001 |
| Minimum prehosp SpO_2_ | 0.988 | 0.976 – 0.999 | 0.04 |

**Additional file 6 – c:** Risk factors associated with death. Among the 13 variables included in the model, only Trauma brain injury, GCS, Lactates, ISS, Minimum SpO_2_ and RIFLE R, I or F were independently associated with mortality. Hosmer and Lemeshow Test (p=0.21). AUC of the model = 0.93 (0.92-0.94). The analysis was conducted on 2362 patients. CI = Confidence Interval, OR = Odds ratio, prehosp=prehospital.
